# Parental income as a marker for socioeconomic position during childhood and later risk of developing a secondary care-diagnosed mental disorder examined across the full diagnostic spectrum: a national cohort study

**DOI:** 10.1186/s12916-020-01794-5

**Published:** 2020-11-16

**Authors:** Christian Hakulinen, Pearl L. H. Mok, Henriette Thisted Horsdal, Carsten B. Pedersen, Preben B. Mortensen, Esben Agerbo, Roger T. Webb

**Affiliations:** 1 Department of Psychology and Logopedics, Faculty of Medicine, University of Helsinki, P.O. Box 21, 00014 Helsinki, Finland; 2 Centre for Pharmacoepidemiology and Drug Safety, Division of Pharmacy and Optometry, Manchester Academic Health Sciences Centre, University of Manchester, Manchester, UK; 3 iPSYCH, The Lundbeck Foundation Initiative for Integrative Psychiatric Research, Aarhus, Denmark; 4 NCRR National Centre for Register-Based Research, School of Business and Social Sciences, Aarhus University, Aarhus V, Denmark; 5 Centre for Integrated Register-Based Research, CIRRAU, Aarhus University, Aarhus, Denmark; 6 Centre for Mental Health and Safety, Division of Psychology and Mental Health, Manchester Academic Health Sciences Centre, University of Manchester, Manchester, UK; 7 NIHR Greater Manchester Patient Safety Translational Research Centre, Manchester, UK

**Keywords:** Socioeconomic position, Income trajectory, Childhood environment, Mental health

## Abstract

**Background:**

Links between parental socioeconomic position during childhood and subsequent risks of developing mental disorders have rarely been examined across the diagnostic spectrum. We conducted a comprehensive analysis of parental income level, including income mobility, during childhood and risks for developing mental disorders diagnosed in secondary care in young adulthood.

**Methods:**

National cohort study of persons born in Denmark 1980–2000 (*N* = 1,051,265). Parental income was measured during birth year and at ages 5, 10 and 15. Follow-up began from 15th birthday until mental disorder diagnosis or 31 December 2016, whichever occurred first. Hazard ratios and cumulative incidence were estimated.

**Results:**

A quarter (25.2%; 95% CI 24.8–25.6%) of children born in the lowest income quintile families will have a secondary care-diagnosed mental disorder by age 37, versus 13.5% (13.2–13.9%) of those born in the highest income quintile. Longer time spent living in low-income families was associated with higher risks of developing mental disorders. Associations were strongest for substance misuse and personality disorders and weaker for mood disorders and anxiety/somatoform disorders. An exception was eating disorders, with low parental income being associated with attenuated risk. For all diagnostic categories examined except for eating disorders, downward socioeconomic mobility was linked with higher subsequent risk and upward socioeconomic mobility with lower subsequent risk of developing mental disorders.

**Conclusions:**

Except for eating disorders, low parental income during childhood is associated with subsequent increased risk of mental disorders diagnosed in secondary care across the diagnostic spectrum. Early interventions to mitigate the disadvantages linked with low income, and better opportunities for upward socioeconomic mobility could reduce social and mental health inequalities.

## Background

Experiencing material deprivation at an early age has been linked with subsequent elevated risk of developing a mental disorder, with children from socially disadvantaged backgrounds being more likely to experience poor mental health in adulthood [1–5]. Although these links have been well-established, much of the published evidence has been generated from cross-sectional studies, and so it remains unclear how risks of mental illnesses could vary in relation to changing parental socioeconomic positions (SEP) during childhood. Researchers have also tended to apply occupational status or educational attainment levels, rather than income levels, as indicators of parental SEP. Whilst the various measures of SEP could reflect different aspects of a child’s home environment, parental income is more likely than parental occupational status or parental educational attainment to fluctuate over time, and could be considered to be a more sensitive measure for capturing any changes in socioeconomic conditions through childhood. Published research on parental SEP and mental health in adulthood has mostly concentrated on specific disorders, mainly depression [6], and it thus remains unclear how risks compare across the whole diagnostic spectrum. In addition, the absolute risks of developing these disorders in relation to childhood socioeconomic conditions have seldom been reported, as previously conducted studies have commonly estimated relative risks only.

Using interlinked national Danish registry data, we conducted a comprehensive longitudinal analysis to explore links between parental income, and income mobility, during childhood and subsequent risk of developing mental disorders that were diagnosed in secondary care settings across the full diagnostic spectrum. Parental income was measured during birth year and at ages 5 (early childhood), 10 (middle childhood) and 15 (adolescence) years. We addressed the following research questions: (1) What are the associations between parental income measured at these four ages and risks of developing any mental disorder and specific disorders after 15th birthday?; (2) Does duration of time spent in materially deprived versus more affluent conditions modify risks?; (3) Are changes in parental income quintile between birth and 15th birthday associated with variability in risk? We examined the following diagnostic groups: (i) any mental disorder, (ii) substance misuse disorders, (iii) personality disorders, (iv) broadly defined schizophrenia, (v) mood disorders, (vi) anxiety/somatoform disorders and (vii) eating disorders. We estimated both relative risks (as hazard ratios) and absolute risks (as cumulative incidence values). Whereas hazard ratios usefully indicate elevations in risk compared to a reference population, absolute risk estimates are more intuitive, clinically relevant and may usefully inform service planning.

## Methods

### Study population

We delineated a national cohort of persons born in Denmark to two Danish-born parents between 1 January 1980 and 31 December 2000, who were living in Denmark at their 15th birthdays. Since 1968, the Danish Civil Registration System [7] has maintained information on all residents, including demographic details and links to parents as well as continuous updates on place of residence and vital status. Unique personal identification numbering enables accurate individual-level interlinkage of multiple national registers. The Danish Data Protection Agency approved the study, with data access agreed by Statistics Denmark and the Danish Health Data Authority. Informed consent from cohort members was not required in this register-based study.

### Parental income measurement

Information about maternal and paternal gross annual income was obtained from the Integrated Database for Labour Market Research [8]. Gross income is a broad income measure, which includes salary, entrepreneurial income, capital income, public transfer payments and pensions, and it was measured in the child’s birth year (age 0) and at ages 5, 10 and 15 years. Parental income was calculated by summing maternal and paternal income. The aggregate values were grouped as quintiles in the national distribution for that calendar year, with quintile 1 representing the lowest income and quintile 5 the highest. Additionally, we constructed a cumulative parental income indicator by adding the parental income quintiles measured at each of the four ages together. This scale ranged from 4 to 20, with a value of 4 representing parental income being consistently in the lowest income quintile across the four ages and a score of 20 indicating living in the most affluent quintile families through childhood. The scale thus indicates both relative parental income levels and their duration between birth and 15th birthday. The scale was applied in two recently reported Danish registry studies [9, 10].

### Classification of mental disorders

Information on psychiatric diagnoses was obtained from the Psychiatric Central Research Register [11]. Using this source, we could examine only persons diagnosed and treated with mental disorders in secondary care settings; i.e. from 1969, patients admitted to an inpatient psychiatric unit or department and, from 1995, those who had contact with a mental health specialist in an outpatient clinic or a general hospital emergency room. The following two groups of mental disorders were not captured in this national register: those diagnosed by general practitioners; those diagnosed by psychiatrists working in private practice, although such practice is rare in Denmark. The Danish modification of the International Classification of Diseases, Eighth Revision (ICD-8) was utilised from 1969 to 1993, with the Tenth Revision (ICD-10) used from 1994 onwards. Classification of the examined mental disorders is shown in Additional file 1: eTable 1.

### Covariates

The following covariates, which were assessed in a previous study [10] and have been linked with elevated risk of developing mental disorders, were fitted in multivariable models: paternal and maternal educational attainment (from the Danish Education Register) [12], history of paternal and maternal mental disorders (from the Danish Psychiatric Central Research Register [11]), degree of urbanisation at cohort members’ place of residence in their birth year [13] and total number of child-parent separation status changes between birth and 15th birthday (from the Danish Civil Registration System [7]). Details of these measures are given in the online supplement (Additional file 1: Additional detailed information on covariates).

### Statistical analysis

Cohort members were followed up from their 15th birthday until first diagnosis of the mental disorder being examined, date of emigration from Denmark, death, or 31 December 2016, whichever came first. For each diagnostic category examined, individuals who had received a relevant diagnosis within that category prior to their 15th birthday were excluded from the analysis. Cox proportional hazard models, adjusted for gender, birth year and time-varying calendar year period, were fitted to estimate the hazard ratios (relative risks) of developing the examined mental disorders. Schoenfeld’s residuals were assessed to verify that the proportional hazards assumption was met. Cumulative incidence (absolute risk) of developing mental disorders from 15th to 37th birthday was estimated using competing-risks regression, [14] with emigration from Denmark and death being treated as competing events. All analyses were conducted using Stata version 15.1.

### Role of the funding source

The funders of the study played no role in the study design, data collection, data analysis, data interpretation, and writing of the manuscript. The corresponding author had full access to all of the study data and had final responsibility for the decision to submit for publication.

## Results

There were 1,051,265 cohort members in 583,005 maternal sibships. Just under a half of all cohort members were women (48.7%), and 107,394 individuals (60.1% women) were diagnosed with a mental disorder in a secondary care setting after reaching age 15 over the aggregated follow-up period of 11.6 million person years. Information pertaining to the distribution of sociodemographic characteristics among cohort members with and without subsequent mental disorder is presented in Additional file 1: eTable 2.

The results presented in Table 1 show an inverse association between parental income quintile at birth and later risk of developing any mental disorder, substance misuse disorders, personality disorders, broadly defined schizophrenia, mood disorders and anxiety/somatoform disorders. These associations were non-linear with considerably higher risks observed among cohort members who were brought up in the poorest families (i.e. income quintile 1). Similar relative risk patterns were observed when parental income at age 15 years was investigated (Additional file 1: eTable 3). In general, risks were higher for substance misuse disorders (quintile 1 at birth: HR 3.44; 95% CI 3.27–3.62), and personality disorders (HR 2.76; 95% CI 2.64–2.88), and somewhat lower for mood disorders (HR 1.72; 95% CI 1.67–1.78) and anxiety/somatoform disorders (HR 2.11; 95% CI 2.06–2.17). Adjustment for additional covariates (parental education, history of parental mental disorder, level of urbanicity at birth and number of changes in child-parental separation status) attenuated the strength of the observed associations, but risk gradients remained. Across the spectrum of mental illnesses, eating disorders was the only diagnostic category for which low parental income did not predict elevated risk. In fact, the risk of developing an eating disorder was lower among cohort members who were in lowest parental income during their birth year (HR 0.90; 95% 0.85–0.96).

**Table 1:** Number of cases, incidence rates and hazard ratios (HRs) for mental disorder diagnostic categories by parental income quintile during birth year

	*n*	Incidence rate	Basic adjustment^a^	Additional adjustment^b^
Any mental disorder				
Q1	30,626	1512	2.09 (2.05, 2.13)	1.52 (1.49, 1.55)
Q2	23,328	1108	1.53 (1.50, 1.56)	1.33 (1.30, 1.36)
Q3	19,853	929	1.28 (1.25, 1.31)	1.20 (1.17, 1.22)
Q4	17,813	826	1.14 (1.11, 1.16)	1.10 (1.08, 1.13)
Q5	15,774	726	1.00 (Ref.)	1.00 (Ref.)
Substance misuse disorder				
Q1	6480	284	3.44 (3.27, 3.62)	1.95 (1.85, 2.07)
Q2	4008	174	2.10 (1.99, 2.22)	1.63 (1.54, 1.72)
Q3	3096	134	1.62 (1.53, 1.72)	1.43 (1.35, 1.52)
Q4	2479	107	1.29 (1.22, 1.37)	1.22 (1.15, 1.30)
Q5	1917	83	1.00 (Ref.)	1.00 (Ref.)
Personality disorders				
Q1	7858	347	2.76 (2.64, 2.88)	1.78 (1.70, 1.87)
Q2	5317	232	1.85 (1.76, 1.93)	1.53 (1.46, 1.60)
Q3	4201	183	1.45 (1.38, 1.52)	1.33 (1.26, 1.39)
Q4	3735	163	1.29 (1.23, 1.36)	1.24 (1.18, 1.30)
Q5	2889	126	1.00 (Ref.)	1.00 (Ref.)
Broadly defined schizophrenia				
Q1	4715	206	2.38 (2.25, 2.50)	1.74 (1.64, 1.84)
Q2	2937	127	1.47 (1.39, 1.55)	1.34 (1.26, 1.42)
Q3	2427	105	1.21 (1.14, 1.28)	1.19 (1.12, 1.26)
Q4	2151	93	1.07 (1.01, 1.14)	1.08 (1.01, 1.15)
Q5	2002	87	1.00 (Ref.)	1.00 (Ref.)
Mood disorders				
Q1	10,482	465	1.72 (1.67, 1.78)	1.32 (1.27, 1.36)
Q2	8497	374	1.38 (1.34, 1.43)	1.23 (1.19, 1.28)
Q3	7256	318	1.18 (1.14, 1.22)	1.12 (1.08, 1.16)
Q4	6629	291	1.08 (1.04, 1.11)	1.05 (1.01, 1.09)
Q5	6151	270	1.00 (Ref.)	1.00 (Ref.)
Anxiety/somatoform disorders				
Q1	18,683	854	2.11 (2.06, 2.17)	1.50 (1.46, 1.54)
Q2	14,109	634	1.57 (1.52, 1.60)	1.36 (1.32, 1.40)
Q3	11,938	533	1.31 (1.28, 1.35)	1.23 (1.20, 1.27)
Q4	10,776	480	1.18 (1.15, 1.22)	1.15 (1.12, 1.18)
Q5	9130	406	1.00 (Ref.)	1.00 (Ref.)
Eating disorders				
Q1	2143	93	0.90 (0.85, 0.96)	0.88 (0.82, 0.93)
Q2	1909	83	0.80 (0.75, 0.85)	0.83 (0.78, 0.89)
Q3	2096	91	0.88 (0.83, 0.93)	0.92 (0.87, 0.98)
Q4	2045	89	0.86 (0.81, 0.92)	0.89 (0.84, 0.94)
Q5	2355	102	1.00 (Ref.)	1.00 (Ref.)

Q = quintile. The reference group is quintile 5 (HR = 1). Incidence rate is reported per 100,000 person years

^a^Basic adjustment—hazard ratios adjusted for gender, birth year and calendar time

^b^Additional adjustment—hazard ratios adjusted for gender, birth year, calendar time, parental mental disorders, parental educational attainment level, degree of urbanicity at birth and number of changes in child-parental separation status between birth and 15th birthday

**Fig. 1:**
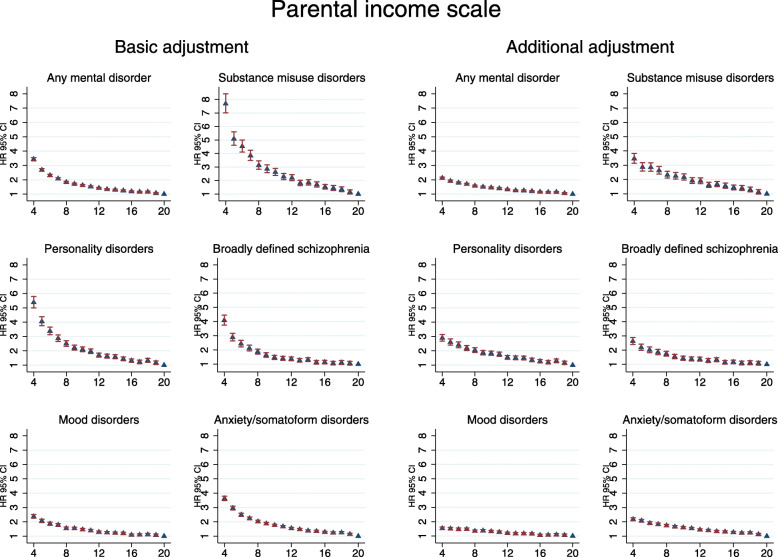
Hazard ratios for any mental disorder, substance misuse disorders, personality disorders, broadly defined schizophrenia, mood disorders, anxiety/somatoform disorders by parental income scale during childhood. The parental income scale represents relative parental income levels and their duration between birth and 15th birthday. A minimum score of 4 represents parental income being in the lowest level across all four age points. A maximum score of 20—the reference category for HRs estimation (HR = 1)—represents parental income being consistently in the highest quintile 5. Basic adjustment—hazard ratios adjusted for gender, birth year and calendar time. Additional adjustment—hazard ratios adjusted for gender, birth year, calendar time, parental mental disorders, parental educational attainment level, degree of urbanicity at birth and number of changes in child-parental separation status between birth and 15th birthday

Figure 1 and Additional file 1: eTable 4 show the associations between duration of parental income levels during childhood, as represented by the cumulative parental income scale, and subsequent risks of developing a mental disorder. The results indicate that children whose families remained in the lowest income quintile through childhood (parental income score = 4) had the highest risk (HR 3.44; 95% CI 3.33–3.55) of developing a mental disorder versus those who experienced affluent conditions throughout (score = 20). Overall, the longer the time a child spent living in a more economically disadvantaged familial environment (i.e. the lower the score), the higher the subsequent risk. Among the diagnostic categories examined (Fig. 1 and Additional file 1: eTables 5–9), the associations were strongest for substance misuse disorders and for personality disorders. Cohort members who remained in the lowest parental income quintile through childhood had much higher risks of developing these conditions versus their peers who were continuously brought up in affluence; substance misuse disorders: HR 7.68; 95% CI 7.01–8.42; personality disorders: HR 5.38; 95% CI 5.00–5.78. On the contrary, although longer time spent living in poorer conditions was still associated with higher risks of developing mood disorders, the associations were much weaker. For all disorders, the associations were attenuated when adjusted for additional covariates, but risk gradients remained (Fig. 1). Cohort members who were in the lowest income quintile in their birth year, and also at ages 5, 10 and 15, had an absolute risk of 34.3% (95% CI 33.4, 35.1%) for being diagnosed with any mental disorder by age 37, compared with 12.0% (95% CI 11.5, 12.5%) risk for those individuals who were in the highest income quintile in their birth years at ages 5, 10 and 15 as well.

**Fig. 2:**
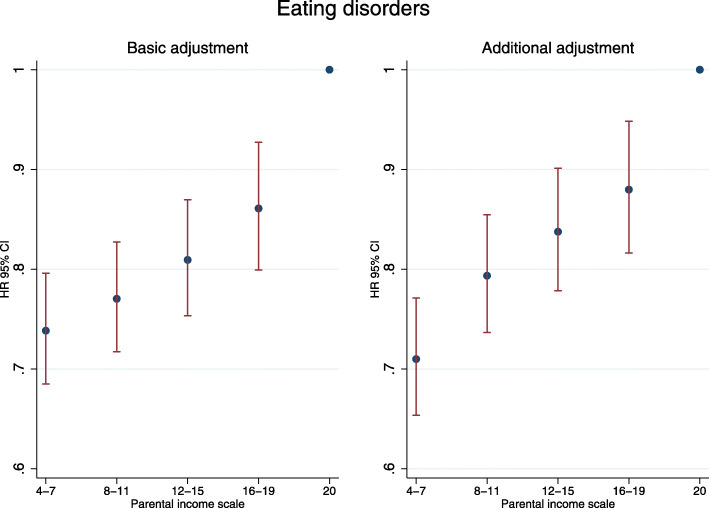
Hazard ratios for eating disorders by parental income scale during childhood. The parental income scale represents relative parental income levels and their duration between birth and 15th birthday. Basic adjustment—hazard ratios adjusted for gender, birth year, and calendar time. Additional adjustment—hazard ratios adjusted for gender, birth year, calendar time, parental mental disorders, parental educational attainment level, degree of urbanicity at birth and number of changes in child-parental separation status between birth and 15th birthday. Parental income scores were aggregated due to small number of cohort members per group

The associations between parental income scale and later risk of developing an eating disorder are shown in Fig. 2. Contrary to the consistent pattern of elevated risk observed in relation to the other mental disorders examined, longer time spent in economically advantaged conditions predicted higher risk for developing an eating disorder. Adjustment for additional covariates did not essentially alter the observed association for this diagnostic category (Fig. 2; Additional file 1: eTable 10).

**Fig. 3:**
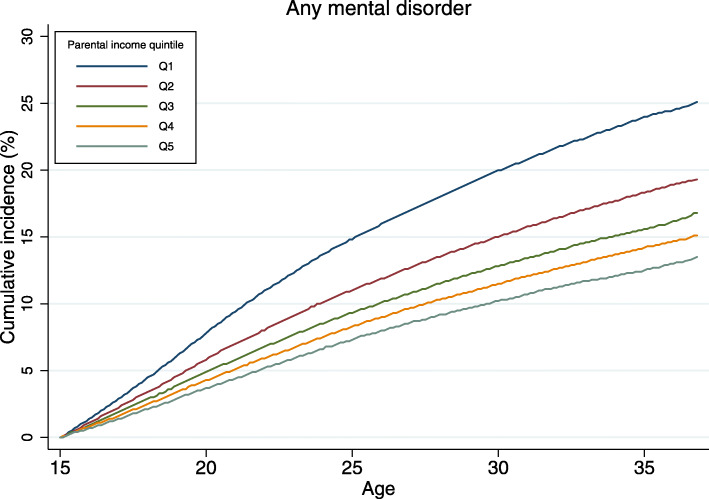
Cumulative incidence of any mental disorder by 37th birthday according to the parental income quintiles during birth year. Q1 is the lowest income (most disadvantaged) quintile whereas Q5 is the highest income (least disadvantaged) quintile. The cumulative incidence percentage value measures the probability of developing any mental disorder before 37th birthday

Cumulative incidence percentage values for developing a mental disorder between 15th and 37th birthday by parental income levels during birth year are shown in Fig. 3. Individuals who were in the lowest income quintile at birth had a 25.2% (95% CI 24.8–25.6%) risk of being diagnosed with a mental disorder by age 37 years, compared with 13.5% (95% CI 13.2–13.9%) for those born in the most affluent quintile. The absolute risks for the specific diagnostic categories examined are shown in Additional file 1: eTable 11. Although relative risks were highest for substance misuse disorders and for personality disorders, children born in the lowest income families had the highest absolute risks of developing anxiety/somatoform disorders (15.8%; 95% CI 15.5–16.2%) and mood disorders (9.6%; 95% CI 9.3–9.9%). Absolute risks were lower for broadly defined schizophrenia (3.9%; 95% CI 3.7–4.0%), substance misuse disorders (5.3%; 95% CI 5.2–5.5%), personality disorders (6.7%; 95% CI 6.5–7.0%) and eating disorders (1.6%; 95% CI 1.5–1.7%).

Risks of developing a mental disorder for the most upwardly mobile cohort members (i.e. children born in quintile 1 and living in quintile 5 at age 15), and for those who experienced the greatest degree of downward mobility through childhood (i.e. born in quintile 5 and living in quintile 1 at age 15), are shown in Table 2. Compared with those who were born and remained in quintile 5 (the most affluent quintile) at age 15, downward mobility was associated with elevated risks of developing mental disorders across the diagnostic spectrum (with the exception of eating disorders). On the contrary, when compared with those who were born and remained in quintile 1 (the lowest quintile) at age 15, upward mobility was associated with decreased risks of developing mental disorders. The exception for this was again eating disorders, for which upward mobility was associated with increased risk.

**Table 2:** Incidence rates and hazard ratios (HRs) for mental disorder diagnostic categories for the most upwardly mobile and the most downwardly mobile cohort members

	*n*	Incidence rate	Basic adjustment^a^	Additional adjustment^b^
Any mental disorder				
The most upwardly mobile (quintile 1 at birth to quintile 5 at age 15)^c^	1640	847.3	0.43 (0.41, 0.45)	0.62 (0.59, 0.66)
The most downwardly mobile (quintile 5 at birth to quintile 1 at age 15)^d^	1331	973.2	1.48 (1.39, 1.57)	1.27 (1.19, 1.35)
Substance misuse disorders				
The most upwardly mobile	204	98.5	0.24 (0.21, 0.28)	0.42 (0.36, 0.49)
The most downwardly mobile	213	143.6	2.22 (1.91, 2.59)	1.74 (1.48, 2.04)
Personality disorders				
The most upwardly mobile	303	146.9	0.30 (0.27, 0.34)	0.49 (0.43, 0.55)
The most downwardly mobile	315	213.6	2.06 (1.82, 2.33)	1.68 (1.48, 1.92)
Broadly defined schizophrenia				
The most upwardly mobile	236	114.1	0.42 (0.37, 0.48)	0.58 (0.51, 0.67)
The most downwardly mobile	184	124.1	1.62 (1.38, 1.90)	1.35 (1.13, 1.60)
Mood disorders				
The most upwardly mobile	669	327.6	0.61 (0.56, 0.66)	0.84 (0.77, 0.91)
The most downwardly mobile	503	343.8	1.36 (1.24, 1.49)	1.15 (1.04, 1.28)
Anxiety/somatoform disorders				
The most upwardly mobile	946	469.2	0.43 (0.40, 0.46)	0.63 (0.59, 0.67)
The most downwardly mobile	817	569.1	1.62 (1.50, 1.74)	1.40 (1.29, 1.51)
Eating disorders				
The most upwardly mobile	222	107.5	1.23 (1.06, 1.43)	1.29 (1.10, 1.50)
The most downwardly mobile	151	101.8	0.92 (0.78, 1.09)	0.90 (0.76, 1.08)

Incidence rate is reported per 100,000 person years

^a^Basic adjustment—hazard ratios adjusted for gender, birth year and calendar time

^b^Additional adjustment—hazard ratios adjusted for gender, birth year, calendar time, parental mental disorders, parental educational attainment level, degree of urbanicity at birth and number of changes in child-parental separation status

^c^The reference group are those persons who were born and remained in quintile 5 at age 15

^d^The reference group are those persons who were born and remained in quintile 1 at age 15

## Discussion

In this cohort study of over a million Danish persons, we found that lower parental income, longer time spent growing up in financially poorer conditions and downward family income mobility during childhood were associated with higher risk for developing a secondary care-diagnosed mental disorder as a young adult. The greatest hazard ratios observed were for substance misuse disorders and for personality disorders, but similar patterns were seen across a broad range of mental disorder diagnostic categories. These risk patterns were attenuated but persisted when further adjustments were made for history of parental mental illness, parental educational attainment levels, urbanicity and number of changes in child-parent separation status between birth and 15th birthday. Importantly, around a quarter of young adults who were born into the lowest income families will have developed a mental disorder by age 37, compared with 13% of those born into the most affluent families. The risk patterns observed were strikingly different for eating disorders, for which lower parental income during childhood was associated with lower subsequent risk of developing disorder.

We conducted a comprehensive investigation of parental income levels during childhood and later risks for developing mental disorders across the full spectrum of psychiatric diagnostic categories. Although there are some notable exceptions [9, 10, 15], most studies that investigated links between parental income and later risks of mental disorder did not take into account changes in income levels during childhood. Using parental income data from early-mid childhood, a study from Sweden has identified five income trajectories and reported that persistence of low family income and downward trajectories in income levels were associated with the highest subsequent risks of developing a range of mental disorders [15]. With a much larger number of cohort members examined in our study, instead of using group-based trajectory modelling, we have taken an optimal alternative approach by investigating temporal fluctuations in parental income levels. We have additionally investigated personality disorders and have for the first time reported absolute risks as well as relative risks. In addition, although it has been established that low SEP in childhood is associated with raised risk of developing mental disorders in adulthood [1–6], most previous studies have applied measures of SEP other than income, or have examined only a small array of mental disorders. The various measures of SEP could reflect different aspects of the childhood living environment, but parental income is likely a more sensitive indicator than the other measures of the available material resources that parents can invest in their children [16]. It does not only represent the financial resources available to the family, but it also acts as a marker for a multitude of unmeasured childhood determinants of later mental disorder, such as neighbourhood environment and housing quality.

We found marked inter-diagnostic differences in the observed associations between parental income during childhood and later risks for developing mental disorders. The associations were stronger for substance misuse disorders and for personality disorders and weaker for mood disorders and for anxiety/somatoform disorders. Although these findings are broadly in line with what has been reported from some previous studies [15], reasons for the diagnostic differences, especially the incongruent associations that we observed for risk of developing an eating disorder, are unclear. Whereas a positive association between higher parental education level and increased risk of eating disorders has previously been reported, findings pertaining to parental income have been mixed for this diagnostic category [15, 17].

Several mechanisms could explain our findings. Low parental income has been linked with other highly detrimental aspects of family environment, including family dysfunctionality, and higher risks of conflicts between family members [18], and of experiencing emotional and physical abuse [19]. Cumulative exposure to adversities in childhood has been shown to be especially harmful not only to later mental health [20], but also to physical health [21]. In our study, the strengths of association were attenuated, although the risk patterns remained essentially similar, after accounting for the potential confounding effects of parental history of mental disorders, parental education attainment level, degree of urbanisation at birth and number of changes in child-parent separation status experienced by cohort members between their births and 15th birthdays. According to social causation theory, exposure to chronic stress caused by lack of socioeconomic resources could at least partly explain why growing up in a more deprived environment is associated with worse health outcomes. Importantly, parental income at birth could also serve as a proxy of the prenatal environment. For example, maternal stress [22] and maternal infections [23] have been associated with increased risk of offspring’s mental disorders. Low parental income thus likely acts as marker for a number of familial and environmental risk factors that are linked with development of later mental disorders through biological and psychosocial mechanisms [24]. Many of these mechanisms could be transdiagnostic—i.e. they increase risk in relation to multiple disorders. However, disorder-specific mechanisms may also be important, especially for eating disorders, for which the risk patterns observed were distinctly different from those of the other diagnostic categories examined. Further investigations are needed to elucidate these complex pathways.

Mental disorders oftentimes are comorbid conditions, and virtually every specific mental disorder is associated with elevated risk of developing every other type of mental disorder across the diagnostic spectrum [25]. Whereas a Danish register-based study estimated that the lifetime prevalence of any secondary care-diagnosed mental disorder is around 33% [26], population-based longitudinal cohort studies suggest that up to 85% of individuals could be diagnosed with a mental disorder at some point during their life [27]. Most mental disorders, however, start developing and are first diagnosed during early adulthood [26–28]. Future studies are needed to estimate whether dynamic socioeconomic conditions in childhood are associated with increased risk of comorbidity, i.e. developing more than one disorder, and longer duration of disorder.

Utilisation of interlinked Danish national registers provided rich information for the entire population including parental income data. Danish psychiatric hospitals and outpatient facilities are freely available to the public, and so access should not be determined by a family’s available financial resources. There is, however, evidence that lower socioeconomic position is associated with less frequent utilisation of mental health services in Denmark [29]. By including only persons born in Denmark with two Danish-born parents, we factored out by design the potential confounding influence of immigrant status on risks of developing a mental disorder [30]. Examining a single study cohort also enabled us to make direct comparisons of risks across a broad range of diagnostic categories without inter-cohort biases. Additionally, the large cohort size and the study’s longitudinal design enabled us to estimate both relative and absolute risks with a high level of statistical power and precision.

Our study also had several limitations. Only individuals diagnosed in secondary care settings were included [31]. Therefore, individuals with milder symptoms who were only treated by a general practitioner or who did not seek help from any type of healthcare service for their mental disorders were not considered. Whereas it can be assumed that more severe disorders, such as schizophrenia, are eventually treated in secondary care and registered, this might be especially problematic for less severe disorders. For example, only a quarter of individuals whose depression was medicated with antidepressants in Denmark were also treated in psychiatric hospital settings for depression [32], indicating that most depression cases are not recorded in the Psychiatric Central Research Register. Adjustment for parental mental illness also likely represented an under-adjustment as most parental psychiatric disorders that were treated in outpatient clinics (without inpatient admission) were not recorded, and all of those that were treated in primary care (without inpatient or outpatient treatment) were unregistered. Furthermore, although register-based mental disorder diagnoses have been shown to have good validity [33, 34], not all mental disorders have been validated. Income from sources that are not officially registered, such as gifts, unreported employment or transfers from parents, were not included. We do not believe that this would have had a marked impact on the observed associations and patterns of risk as illicit work is relatively uncommon in Denmark compared to other European countries [35]. Detailed information on psychosocial factors, which could help to explain the link between parental income during childhood and later risk of developing a mental disorder, is also not recorded in these administrative registers. Thus, the possibility of residual confounding cannot be ruled out in observational studies such as ours, and results should be interpreted with caution. Last, no correction of standard errors for sibship clustering was performed.

It is important to note that rather than investigating the causal effect of parental income per se, we conceptualised parental income as an indicator for an array of measured and unmeasured monetary and non-monetary indices of family’s social environments that are typically interrelated. As our results indicate, controlling for a range of covariates attenuates the relative risk estimates observed, but such estimates would still only constitute partial adjustments as it would never be possible to control for all relevant correlates, and therefore completely disentangle these non-monetary influences from the direct monetary impact. It is also plausible to posit that our adjusted estimates are actually over-adjusted, as some of the adjusted variables likely lie on the causal pathway between parental income levels and trajectories through childhood and subsequent risks for developing mental disorders as a young adult. For example, parental educational attainment is likely a key causal determinant of parental income level and thus adjusting for it as a potential confounder may inappropriately remove some of the association that we seek to observe.

Income inequality and the prevalence of childhood poverty in Denmark are ranked as being among the lowest among countries worldwide [36]. The income quintile share ratio is a measure of income equality and is defined as “the ratio of total income received by the 20 % of the population with the highest income (top quintile) to that received by the 20 % of the population with the lowest income (lowest quintile)”; in 2016, the ratio was 4.06 in Denmark versus the EU average of 5.16 [37]. The associations observed in this study may therefore be even more pronounced in other countries that have greater levels of income disparities and other social inequalities, where universal healthcare provision is lacking, and where welfare benefits for disadvantaged individuals and families are more limited.

## Conclusions

Our study shows how dynamic socioeconomic conditions in childhood are associated with elevated risks for developing a broad range of mental disorders diagnosed in secondary care settings and that longer time spent growing up in financially poorer conditions may be especially harmful to later mental health. Interventions to mitigate the disadvantages linked with low income, and better opportunities for upward socioeconomic mobility, are needed to reduce social inequality in mental health, which in turn could lower the risk of developing socioeconomically patterned somatic conditions in later life [38]. Since longer time spent in low-income conditions was linked with higher subsequent risks of developing mental illnesses, preventive measures targeted at early childhood could be especially beneficial in reducing these enduring risks.

## Supplementary information

**Additional file 1: **Additional detailed information on covariates. **eTables 1–11**. **eTable 1** - Classification of the mental disorder diagnostic categories. **eTable 2** - Sociodemographic characteristics of individuals according to any mental disorder diagnosis. **eTable 3** - Number of cases, incidence rates and hazard ratios (HRs) for any mental disorder and for each diagnostic category by parental income quintile at age 15 years. **eTable 4** - Hazard ratios for developing any mental disorder by cumulative parental income scale during childhood. **eTable 5** - Hazard ratios for developing a substance misuse disorder by cumulative parental income scale during childhood. **eTable 6** - Hazard ratios for developing a personality disorder by cumulative parental income scale during childhood. **eTable 7** - Hazard ratios for developing broadly defined schizophrenia by cumulative parental income scale during childhood. **eTable 8** - Hazard ratios for developing a mood disorder by cumulative parental income scale during childhood. **eTable 9** - Hazard ratios for developing an anxiety/somatoform disorder by cumulative parental income scale during childhood. **eTable 10** - Hazard ratios for developing an eating disorder by cumulative parental income scale during childhood. **eTable 11** - Cumulative incidence for developing any mental disorder and for each diagnostic category at age 37 years by parental income quintile during birth-year.

## Data Availability

The data that support the findings of this study are available from Statistics Denmark. Restrictions apply to the availability of these data, which were used under license for this study. For information on accessing the data, see www.dst.dk.
