# Supplementary material for: Parental income as a marker for socioeconomic position during childhood and later risk of developing a secondary care-diagnosed mental disorder examined across the full diagnostic spectrum: a national cohort study
Source: BMC Med. 2020 Nov 16;18:323. doi: 10.1186/s12916-020-01794-5 (PMC7667856; doi:10.1186/s12916-020-01794-5)
Supplement: Supplementary file 1 — Additional file 1: Additional detailed information on covariates. eTables 1–11. eTable 1 - Classification of the mental disorder diagnostic categories. eTable 2 - Sociodemographic characteristics of individuals according to any mental disorder diagnosis. eTable 3 - Number of cases, incidence rates and hazard ratios (HRs) for any mental disorder and for each diagnostic category by parental income quintile at age 15 years. eTable 4 - Hazard ratios for developing any mental disorder by cumulative parental income scale during childhood. eTable 5 - Hazard ratios for developing a substance misuse disorder by cumulative parental income scale during childhood. eTable 6 - Hazard ratios for developing a personality disorder by cumulative parental income scale during childhood. eTable 7 - Hazard ratios for developing broadly defined schizophrenia by cumulative parental income scale during childhood. eTable 8 - Hazard ratios for developing a mood disorder by cumulative parental income scale during childhood. eTable 9 - Hazard ratios for developing an anxiety/somatoform disorder by cumulative parental income scale during childhood. eTable 10 - Hazard ratios for developing an eating disorder by cumulative parental income scale during childhood. eTable 11 - Cumulative incidence for developing any mental disorder and for each diagnostic category at age 37 years by parental income quintile during birth-year. [file 12916_2020_1794_MOESM1_ESM.docx]

**Parental income during childhood and later risk of developing a secondary care-diagnosed mental disorder examined across the full diagnostic spectrum: a national cohort study**

**ONLINE SUPPLEMENT APPENDIX**

**Additional detailed information on covariates**

**eTable 1. Classification of the mental disorder diagnostic categories**

**eTable 2. Sociodemographic characteristics of individuals according to any mental disorder diagnosis**

**eTable 3. Number of cases, incidence rates and hazard ratios (HRs) for any mental disorder and for each diagnostic category by parental income quintile at age 15 years**

**eTable 4. Hazard ratios for developing any mental disorder by cumulative parental income scale during childhood**

**eTable 5. Hazard ratios for developing a substance misuse disorder by cumulative parental income scale during childhood**

**eTable 6. Hazard ratios for developing a personality disorder by cumulative parental income scale during childhood**

**eTable 7. Hazard ratios for developing broadly defined schizophrenia by cumulative parental income scale during childhood**

**eTable 8. Hazard ratios for developing a mood disorder by cumulative parental income scale during childhood**

**eTable 9. Hazard ratios for developing an anxiety/somatoform disorder by cumulative parental income scale during childhood**

**eTable 10. Hazard ratios for developing an eating disorder by cumulative parental income scale during childhood**

**eTable 11. Cumulative incidence for developing any mental disorder and for each diagnostic category at age 37 years by parental income quintile during birth-year**

**Additional detailed information on covariates**

**History of paternal and maternal mental disorders**

The Psychiatric Central Register^1^ contains information on all inpatient psychiatric admissions from 1969, and all outpatient and emergency department visits from 1995. Danish modification of the International Classification of Diseases, 8^th^ Revision (ICD-8)^2^ was used from 1969 to 1993, and the ICD-10 Classification of Mental and Behavioural Disorders: Diagnostic Criteria for Research (ICD-10-DCR) has been in use since 1994.^3^ Paternal and maternal mental disorders were classified into two groups (1 = a history of secondary-care treated mental disorders; 0 = no history of secondary-care treated mental disorders)^4^, and fitted as separate covariates in the multivariable models.

**Paternal and maternal educational attainment level**

Population Education Register^5^ contains information on completed educational level. Paternal and maternal education was extracted and categorised into two groups: 0 = no secondary or higher education; 1 = any secondary or higher education. Paternal and maternal educational attainment levels were fitted as separate covariates in the multivariable models.

**Degree of urbanisation of cohort member’s residence in year of birth**

This information was acquired from the Civil Registration System^6^, and it was groups as follows: capital, capital suburb, provincial city, provincial town, or rural area.^7^

**Total number of changes in child-parent separation status**

The Civil Registration System^6^ contains information of residential address of individuals living in Denmark. Child-parent separation was defined according to cohort members residence status, i.e., whether they were residing or not residing with their legal parents at birth and at each birthday from 1^st^ to 15^th^, inclusive. Separation status was categorised as follows: no separation (living with both parents); paternal separation (living with the mother, but not father); maternal separation (living with father, but not mother); maternal and paternal separation (living with neither parent); or missing (unknown).^8^ The missing category included individuals with missing child-parent separation status information for at least one year. ﻿The total number of changes in child-parent separation status during the 15-year period from birth to 15^th^ birthday was categorised as 0, 1, 2, 3, 4, and 5 or more number of changes.^9^

**References**

1. Mors O, Perto GP, Mortensen PB. The Danish Psychiatric Central Research Register. *Scand J Public Health*. 2011;39(7 Suppl):54-57.
2. World Health Organization. Classification of Diseases: Extended Danish-Latin Version of the World Health Organization International Classification of Diseases, 8th Revision, 1965. Copenhagen: Danish National Board of Health; 1971.
3. World Health Organization. The ICD-10 Classification of Mental and Behavioural Disorders: Diagnostic Criteria for Research. Geneva, Switzerland: World Health Organization; 1993.
4. Pedersen CB, Mors O, Bertelsen A, et al. A comprehensive nationwide study of the incidence rate and lifetime risk for treated mental disorders. *JAMA Psychiatry*. 2014;71(5):573-581.
5. Jensen VM, Rasmussen AW. Danish Education Registers. *Scand J Public Health*. 2011;39(7 Suppl):91-94.
6. Pedersen CB. The Danish Civil Registration System. *Scand J Public Health*. 2011;39(7 Suppl):22-25.
7. Vassos E, Agerbo E, Mors O, Bøcker Pedersen C. Urban-rural differences in incidence rates of psychiatric disorders in Denmark. *Br J Psychiatry*. 2016;208(5):435-440.
8. Paksarian D, Eaton WW, Mortensen PB, Merikangas KR, Pedersen CB. A population-based study of the risk of schizophrenia and bipolar disorder associated with parent-child separation during development. *Psychol Med*. 2015;45(13):2825-2837.
9. Astrup A, Pedersen CB, Mok PLH, Carr MJ, Webb RT. Self-harm risk between adolescence and midlife in people who experienced separation from one or both parents during childhood. *J Affect Disord*. 2017;208:582-589.

**eTable 1. Classification of the mental disorder diagnostic categories**

| **Diagnosis** | **ICD-10 codes** | **Equivalent ICD-8 codes** |
| --- | --- | --- |
| Any mental disorder | F00-F99 | 290-315 |
| Substance misuse disorder | F10-F19 | 291.x9, 294.39, 303.x9, 303.20, 303.28, 303.90, 304.x9 |
| Broadly defined schizophrenia | F20-F29 | 295.x9, 296.89, 297.x9, 298.29-298.99, 299.04, 299.05, 299.09, 301.83 |
| Mood disorders | F30-F39 | 296.x9 (excluding 296.89), 298.09, 298.19, 300.49, 301.19 |
| Anxiety/somatoform disorder | F40-F48 | 300.x9 (excluding 300.49), 305.x9, 305.68, 307.99 |
| Eating disorders | F50 | 305.60, 306.50, 306.58, 306.59 |
| Personality disorders | F60 | 301.x9 (excluding 301.19), 301.80, 301.81, 301.82, 301.84 |

**eTable 2.** **Sociodemographic characteristics of cohort members according to any mental disorder diagnosis**

|  | **Any mental disorder** | |
| --- | --- | --- |
|  | **Yes (n=107,394)** | **No (n=900,858)** |
| Male | 42,823 (39.9%) | 470,242 (52.2%) |
| Female | 64,571 (60.1%) | 430,616 (47.8%) |
|  |  |  |
| **History of paternal mental disorders** |  |  |
| No history | 90,608 (84.4%) | 831,085 (92.3%) |
| With a history | 16,786 (15.6%) | 69,773 (7.7%) |
|  |  |  |
| **History of maternal mental disorders** |  |  |
| No history | 85,810 (79.9%) | 816,609 (90.6%) |
| With a history | 21,584 (20.1%) | 84,249 (9.4%) |
|  |  |  |
| **Paternal educational attainment** |  |  |
| Any secondary education | 71,164 (66.3%) | 682,408 (75.8%) |
| No secondary education | 33,482 (31.2%) | 204,568 (22.7%) |
| Missing information | 2748 (2.6%) | 13,882 (1.5%) |
|  |  |  |
| **Maternal educational attainment** |  |  |
| Any secondary education | 70,204 (65.4%) | 682,653 (75.8%) |
| No secondary education | 35,927 (33.5%) | 211,565 (23.5%) |
| Missing information | 1263 (1.2%) | 6640 (0.7%) |
|  |  |  |
| **Degree of urbanisation of residence during birth-year** |  |  |
| Capital | 11,157 (10.4%) | 91,420 (10.1%) |
| Capital suburb | 14,656 (13.6%) | 111,657 (12.4%) |
| Provincial city | 12,548 (11.7%) | 105,290 (11.7%) |
| Provincial town | 30,586 (28.5%) | 250,950 (27.9%) |
| Rural area | 38,445 (35.8%) | 341,520 (37.9%) |
|  |  |  |
| **Total no. of changes in child-parent separation status** |  |  |
| 0 | 54,210 (50.5%) | 593,539 (65.9%) |
| 1 | 29,666 (27.6%) | 190,532 (21.2%) |
| 2 | 10,043 (9.4%) | 52,222 (5.8%) |
| 3 | 5706 (5.3%) | 24,366 (2.7%) |
| 4 | 2188 (2%) | 7425 (0.8%) |
| 5 or more | 1687 (1.6%) | 4947 (0.5%) |
| Missing information | 3894 (3.6%) | 27,827 (3.1%) |

Of the 1,051,265 cohort members, 43,013 persons were diagnosed with a mental disorder in secondary care setting prior to their 15th birthday and were excluded from the analysis of any mental disorder.

**eTable 3. Number of cases, incidence rates and hazard ratios (HRs) for any mental disorder and for each diagnostic category by parental income quintile at age 15 years**

|  | **N** | **Incidence rate** | **Basic adjustment^a^** | **Additional adjustment^b^** |
| --- | --- | --- | --- | --- |
| Any mental disorder |  |  |  |  |
| Q1 | 31,811 | 1583 | 2.30 (2.26, 2.35) | 1.65 (1.61, 1.68) |
| Q2 | 23,081 | 1096 | 1.59 (1.56, 1.62) | 1.40 (1.37, 1.43) |
| Q3 | 19,825 | 926 | 1.34 (1.31, 1.37) | 1.26 (1.24, 1.29) |
| Q4 | 17,579 | 814 | 1.18 (1.15, 1.20) | 1.15 (1.13, 1.18) |
| Q5 | 15,098 | 692 | 1.00 (Ref.) | 1.00 (Ref.) |
| Substance misuse disorder |  |  |  |  |
| Q1 | 6847 | 300 | 3.99 (3.79, 4.21) | 2.28 (2.15, 2.41) |
| Q2 | 3965 | 172 | 2.29 (2.16, 2.42) | 1.82 (1.72, 1.93) |
| Q3 | 2979 | 129 | 1.71 (1.61, 1.82) | 1.53 (1.44, 1.63) |
| Q4 | 2445 | 106 | 1.40 (1.32, 1.49) | 1.35 (1.27, 1.44) |
| Q5 | 1744 | 76 | 1.00 (Ref.) | 1.00 (Ref.) |
| Personality disorders |  |  |  |  |
| Q1 | 8520 | 376 | 3.29 (3.15, 3.43) | 2.11 (2.01, 2.22) |
| Q2 | 5275 | 230 | 2.01 (1.92, 2.10) | 1.68 (1.60, 1.77) |
| Q3 | 4112 | 179 | 1.56 (1.49, 1.64) | 1.44 (1.37, 1.51) |
| Q4 | 3449 | 150 | 1.30 (1.24, 1.37) | 1.26 (1.20, 1.33) |
| Q5 | 2644 | 115 | 1.00 (Ref.) | 1.00 (Ref.) |
| Broadly defined schizophrenia |  |  |  |  |
| Q1 | 4773 | 208 | 2.58 (2.44, 2.72) | 1.84 (1.74, 1.96) |
| Q2 | 2985 | 129 | 1.60 (1.51, 1.70) | 1.48 (1.39, 1.57) |
| Q3 | 2425 | 105 | 1.30 (1.22, 1.38) | 1.28 (1.21, 1.36) |
| Q4 | 2182 | 94 | 1.17 (1.10, 1.24) | 1.19 (1.11, 1.26) |
| Q5 | 1867 | 81 | 1.00 (Ref.) | 1.00 (Ref.) |
| Mood disorders |  |  |  |  |
| Q1 | 10,690 | 474 | 1.81 (1.76, 1.87) | 1.35 (1.30, 1.40) |
| Q2 | 8305 | 365 | 1.40 (1.35, 1.44) | 1.25 (1.21, 1.29) |
| Q3 | 7344 | 322 | 1.23 (1.19, 1.27) | 1.17 (1.13, 1.21) |
| Q4 | 6696 | 294 | 1.12 (1.08, 1.16) | 1.10 (1.06, 1.14) |
| Q5 | 5980 | 262 | 1.00 (Ref.) | 1.00 (Ref.) |
| Anxiety/somatoform disorders |  |  |  |  |
| Q1 | 19,833 | 910 | 2.43 (2.37, 2.49) | 1.71 (1.66-1.76) |
| Q2 | 14,020 | 630 | 1.68 (1.63, 1.72) | 1.47 (1.43-1.52) |
| Q3 | 11,936 | 533 | 1.42 (1.38, 1.46) | 1.34 (1.30-1.38) |
| Q4 | 10,328 | 459 | 1.22 (1.18, 1.25) | 1.20 (1.16-1.23) |
| Q5 | 8519 | 377 | 1.00 (Ref.) | 1.00 (Ref.) |
| Eating disorders |  |  |  |  |
| Q1 | 2004 | 87 | 0.83 (0.78, 0.88) | 0.80 (0.75, 0.86) |
| Q2 | 1943 | 84 | 0.81 (0.76, 0.86) | 0.83 (0.78, 0.88) |
| Q3 | 2001 | 87 | 0.83 (0.78, 0.88) | 0.86 (0.81, 0.91) |
| Q4 | 2201 | 96 | 0.91 (0.86, 0.97) | 0.93 (0.88, 0.98) |
| Q5 | 2399 | 104 | 1.00 (Ref.) | 1.00 (Ref.) |

Q=quintile. The reference group is quintile 5 (HR=1). Incidence rate is reported per 100,000 person years.

^a^ Basic adjustment - hazard ratios adjusted for gender, birth-year, and calendar time.

^b^ Additional adjustment - hazard ratios adjusted for gender, birth-year, calendar time, parental mental disorders, parental educational attainment level, degree of urbanicity at birth, and number of changes in child-parental separation status between birth and 15^th^ birthday.

**eTable 4. Hazard ratios for developing any mental disorder by cumulative parental income scale during childhood**

| **Cumulative parental income scale** | **Hazard ratios (95% CI)** | |
| --- | --- | --- |
|  | **Basic adjustment ^a^** | **Additional adjustment ^b^** |
| 4 | 3.44 (3.33, 3.55) | 2.12 (2.05, 2.20) |
| 5 | 2.68 (2.59, 2.78) | 1.91 (1.84, 1.98) |
| 6 | 2.31 (2.23, 2.39) | 1.79 (1.72, 1.85) |
| 7 | 2.08 (2.01, 2.15) | 1.70 (1.64, 1.76) |
| 8 | 1.83 (1.77, 1.89) | 1.57 (1.52, 1.63) |
| 9 | 1.71 (1.65, 1.77) | 1.50 (1.45, 1.56) |
| 10 | 1.61 (1.55, 1.67) | 1.45 (1.40, 1.50) |
| 11 | 1.52 (1.46, 1.57) | 1.39 (1.34, 1.44) |
| 12 | 1.42 (1.37, 1.47) | 1.32 (1.28, 1.37) |
| 13 | 1.33 (1.28, 1.38) | 1.25 (1.21, 1.30) |
| 14 | 1.30 (1.25, 1.35) | 1.25 (1.20, 1.29) |
| 15 | 1.24 (1.20, 1.29) | 1.20 (1.15, 1.25) |
| 16 | 1.18 (1.14, 1.23) | 1.15 (1.11, 1.20) |
| 17 | 1.16 (1.11, 1.20) | 1.14 (1.09, 1.19) |
| 18 | 1.16 (1.11, 1.21) | 1.14 (1.09, 1.19) |
| 19 | 1.06 (1.02, 1.11) | 1.05 (1.01, 1.10) |
| 20 | 1.00 (Ref.) | 1.00 (Ref.) |

^a^ Basic adjustment - hazard ratios adjusted for gender, birth-year, and calendar time

^b^ Additional adjustment - hazard ratios adjusted for gender, birth-year, calendar time, parental mental disorders, parental educational attainment level, degree of urbanicity at birth, and number of changes in child-parental separation status between birth and 15^th^ birthday.

**eTable 5. Hazard ratios for developing a substance misuse disorder by cumulative parental income scale during childhood**

| **Cumulative parental income scale** | **Hazard ratios (95% CI)** | |
| --- | --- | --- |
|  | **Basic adjustment ^a^** | **Additional adjustment ^b^** |
| 4 | 7.68 (7.01, 8.42) | 3.46 (3.14, 3.82) |
| 5 | 5.08 (4.61, 5.61) | 2.87 (2.59, 3.18) |
| 6 | 4.53 (4.11, 4.99) | 2.86 (2.58, 3.16) |
| 7 | 3.85 (3.49, 4.24) | 2.63 (2.38, 2.91) |
| 8 | 3.13 (2.84, 3.45) | 2.32 (2.10, 2.57) |
| 9 | 2.85 (2.58, 3.15) | 2.25 (2.03, 2.49) |
| 10 | 2.62 (2.37, 2.89) | 2.17 (1.95, 2.40) |
| 11 | 2.28 (2.06, 2.53) | 1.91 (1.72, 2.12) |
| 12 | 2.20 (1.98, 2.44) | 1.90 (1.71, 2.11) |
| 13 | 1.81 (1.62, 2.02) | 1.62 (1.45, 1.80) |
| 14 | 1.85 (1.65, 2.06) | 1.67 (1.50, 1.87) |
| 15 | 1.70 (1.52, 1.90) | 1.56 (1.39, 1.75) |
| 16 | 1.52 (1.36, 1.71) | 1.44 (1.28, 1.61) |
| 17 | 1.44 (1.27, 1.63) | 1.39 (1.23, 1.58) |
| 18 | 1.34 (1.17, 1.53) | 1.29 (1.13, 1.47) |
| 19 | 1.14 (0.99, 1.31) | 1.12 (0.98. 1.29) |
| 20 | 1.00 (Ref.) | 1.00 (Ref.) |

^a^ Basic adjustment - hazard ratios adjusted for gender, birth-year, and calendar time

^b^ Additional adjustment - hazard ratios adjusted for gender, birth-year, calendar time, parental mental disorders, parental educational attainment level, degree of urbanicity at birth, and number of changes in child-parental separation status between birth and 15^th^ birthday.

**eTable 6. Hazard ratios for developing a personality disorder by cumulative parental income scale during childhood**

| **Cumulative parental income scale** | **Hazard ratios (95% CI)** | |
| --- | --- | --- |
|  | **Basic adjustment ^a^** | **Additional adjustment ^b^** |
| 4 | 5.38 (5.00, 5.78) | 2.89 (2.67, 3.13) |
| 5 | 4.05 (3.74, 4.38) | 2.60 (2.40, 2.83) |
| 6 | 3.38 (3.12, 3.65) | 2.41 (2.22, 2.62) |
| 7 | 2.87 (2.65, 3.10) | 2.19 (2.01, 2.37) |
| 8 | 2.50 (2.31, 2.71) | 2.04 (1.88, 2.21) |
| 9 | 2.21 (2.04, 2.40) | 1.86 (1.72, 2.02) |
| 10 | 2.10 (1.94, 2.27) | 1.82 (1.68, 1.98) |
| 11 | 1.96 (1.81, 2.13) | 1.75 (1.61, 1.90) |
| 12 | 1.69 (1.55, 1.84) | 1.53 (1.41, 1.67) |
| 13 | 1.62 (1.49, 1.77) | 1.50 (1.38, 1.64) |
| 14 | 1.58 (1.44, 1.72) | 1.49 (1.37, 1.63) |
| 15 | 1.43 (1.31, 1.57) | 1.35 (1.24, 1.49) |
| 16 | 1.31 (1.19, 1.43) | 1.27 (1.16, 1.39) |
| 17 | 1.22 (1.10, 1.35) | 1.20 (1.08, 1.32) |
| 18 | 1.33 (1.20, 1.47) | 1.30 (1.17, 1.44) |
| 19 | 1.16 (1.05, 1.29) | 1.15 (1.04, 1.28) |
| 20 | 1.00 (Ref.) | 1.00 (Ref.) |

^a^ Basic adjustment - hazard ratios adjusted for gender, birth-year, and calendar time

^b^ Additional adjustment - hazard ratios adjusted for gender, birth-year, calendar time, parental mental disorders, parental educational attainment level, degree of urbanicity at birth, and number of changes in child-parental separation status between birth and 15^th^ birthday.

**eTable 7. Hazard ratios for developing broadly defined schizophrenia by cumulative parental income scale during childhood**

| **Cumulative parental income scale** | **Hazard ratios (95% CI)** | |
| --- | --- | --- |
|  | **Basic adjustment ^a^** | **Additional adjustment ^b^** |
| 4 | 4.09 (3.75, 4.46) | 2.63 (2.39, 2.89) |
| 5 | 2.89 (2.63, 3.18) | 2.19 (1.98, 2.43) |
| 6 | 2.43 (2.21, 2.68) | 2.02 (1.83, 2.23) |
| 7 | 2.12 (1.93, 2.34) | 1.86 (1.68, 2.05) |
| 8 | 1.87 (1.71, 2.06) | 1.74 (1.58, 1.92) |
| 9 | 1.63 (1.48, 1.80) | 1.55 (1.40, 1.71) |
| 10 | 1.47 (1.33, 1.62) | 1.42 (1.29, 1.58) |
| 11 | 1.40 (1.26, 1.54) | 1.38 (1.24, 1.52) |
| 12 | 1.37 (1.24, 1.51) | 1.36 (1.23, 1.51) |
| 13 | 1.26 (1.14, 1.40) | 1.26 (1.14, 1.41) |
| 14 | 1.31 (1.18, 1.45) | 1.32 (1.19, 1.47) |
| 15 | 1.12 (1.00, 1.25) | 1.14 (1.02, 1.28) |
| 16 | 1.14 (1.02, 1.27) | 1.16 (1.04, 1.30) |
| 17 | 1.06 (0.94, 1.19) | 1.09 (0.97, 1.23) |
| 18 | 1.10 (0.97, 1.25) | 1.11 (0.98, 1.26) |
| 19 | 1.05 (0.93, 1.19) | 1.08 (0.95, 1.22) |
| 20 | 1.00 (Ref.) | 1.00 (Ref.) |

^a^ Basic adjustment - hazard ratios adjusted for gender, birth-year, and calendar time

^b^ Additional adjustment - hazard ratios adjusted for gender, birth-year, calendar time, parental mental disorders, parental educational attainment level, degree of urbanicity at birth, and number of changes in child-parental separation status between birth and 15^th^ birthday.

**eTable 8. Hazard ratios for developing a mood disorder by cumulative parental income scale during childhood**

| **Cumulative parental income scale** | **Hazard ratios (95% CI)** | |
| --- | --- | --- |
|  | **Basic adjustment ^a^** | **Additional adjustment ^b^** |
| 4 | 2.37 (2.25, 2.50) | 1.53 (1.44, 1.62) |
| 5 | 2.06 (1.95, 2.18) | 1.51 (1.42, 1.61) |
| 6 | 1.86 (1.76, 1.97) | 1.47 (1.38, 1.56) |
| 7 | 1.78 (1.68, 1.88) | 1.48 (1.39, 1.56) |
| 8 | 1.55 (1.46, 1.64) | 1.34 (1.27, 1.42) |
| 9 | 1.54 (1.46, 1.63) | 1.37 (1.29, 1.45) |
| 10 | 1.45 (1.37, 1.54) | 1.32 (1.24, 1.39) |
| 11 | 1.38 (1.31, 1.46) | 1.27 (1.20, 1.35) |
| 12 | 1.28 (1.21, 1.36) | 1.19 (1.13, 1.27) |
| 13 | 1.24 (1.17, 1.32) | 1.17 (1.10, 1.24) |
| 14 | 1.22 (1.14, 1.29) | 1.16 (1.10, 1.24) |
| 15 | 1.20 (1.12, 1.27) | 1.15 (1.08, 1.22) |
| 16 | 1.08 (1.02, 1.15) | 1.06 (0.99, 1.12) |
| 17 | 1.09 (1.02, 1.16) | 1.06 (0.99, 1.14) |
| 18 | 1.11 (1.03, 1.19) | 1.09 (1.01, 1.17) |
| 19 | 1.07 (1.00, 1.15) | 1.06 (0.99, 1.14) |
| 20 | 1.00 (Ref.) | 1.00 (Ref.) |

^a^ Basic adjustment - hazard ratios adjusted for gender, birth-year, and calendar time

^b^ Additional adjustment - hazard ratios adjusted for gender, birth-year, calendar time, parental mental disorders, parental educational attainment level, degree of urbanicity at birth, and number of changes in child-parental separation status between birth and 15^th^ birthday.

**eTable 9. Hazard ratios for developing an anxiety/somatoform disorder by cumulative parental income scale during childhood**

| **Cumulative parental income scale** | **Hazard ratios (95% CI)** | |
| --- | --- | --- |
|  | **Basic adjustment ^a^** | **Additional adjustment ^b^** |
| 4 | 3.63 (3.48, 3.79) | 2.18 (2.08, 2.28) |
| 5 | 2.95 (2.82, 3.09) | 2.07 (1.97, 2.17) |
| 6 | 2.49 (2.37, 2.61) | 1.90 (1.81, 2.00) |
| 7 | 2.24 (2.14, 2.34) | 1.83 (1.74, 1.91) |
| 8 | 2.02 (1.93, 2.11) | 1.74 (1.66, 1.82) |
| 9 | 1.87 (1.79, 1.96) | 1.65 (1.58, 1.73) |
| 10 | 1.76 (1.68, 1.84) | 1.60 (1.52, 1.67) |
| 11 | 1.66 (1.59, 1.74) | 1.54 (1.47, 1.62) |
| 12 | 1.54 (1.47, 1.62) | 1.44 (1.38, 1.52) |
| 13 | 1.47 (1.40, 1.54) | 1.40 (1.33, 1.47) |
| 14 | 1.37 (1.31, 1.45) | 1.33 (1.26, 1.40) |
| 15 | 1.34 (1.28, 1.41) | 1.30 (1.24, 1.37) |
| 16 | 1.28 (1.22, 1.34) | 1.26 (1.20, 1.33) |
| 17 | 1.23 (1.16, 1.30) | 1.22 (1.15, 1.29) |
| 18 | 1.24 (1.17, 1.32) | 1.23 (1.16, 1.30) |
| 19 | 1.13 (1.07, 1.20) | 1.13 (1.06, 1.20) |
| 20 | 1.00 (Ref.) | 1.00 (Ref.) |

^a^ Basic adjustment - hazard ratios adjusted for gender, birth-year, and calendar time

^b^ Additional adjustment - hazard ratios adjusted for gender, birth-year, calendar time, parental mental disorders, parental educational attainment level, degree of urbanicity at birth, and number of changes in child-parental separation status between birth and 15^th^ birthday.

**eTable 10. Hazard ratios for developing an eating disorder by cumulative parental income scale during childhood**

| **Cumulative parental income scale** | **Hazard ratios (95% CI)** | |
| --- | --- | --- |
|  | **Basic adjustment ^a^** | **Additional adjustment ^b^** |
| 4-7 | 0.74 (0.69, 0.80) | 0.71 (0.65, 0.77) |
| 8-11 | 0.77 (0.72, 0.83) | 0.79 (0.74, 0.85) |
| 12-15 | 0.81 (0.75, 0.87) | 0.84 (0.78, 0.90) |
| 16-19 | 0.86 (0.80, 0.93) | 0.88 (0.82, 0.95) |
| 20 | 1.00 (Ref.) | 1.00 (Ref.) |

Parental income scores were aggregated due to small number of cohort members per group.

^a^ Basic adjustment - hazard ratios adjusted for gender, birth-year, and calendar time

^b^ Additional adjustment - hazard ratios adjusted for gender, birth-year, calendar time, parental mental disorders, parental educational attainment level, degree of urbanicity at birth, and number of changes in child-parental separation status between birth and 15^th^ birthday.

**eTable 11. Cumulative incidence for developing any mental disorder and for each diagnostic category at age 37 years by parental income quintile during birth-year**

|  | **Cumulative incidence % (95% CI)** |
| --- | --- |
| Any mental disorder |  |
| Q1 | 25.2 (24.8, 25.6) |
| Q2 | 19.4 (19.0, 19.7) |
| Q3 | 16.8 (16.5, 17.1) |
| Q4 | 15.2 (14.9, 15.6) |
| Q5 | 13.5 (13.2, 13.9) |
| Substance misuse disorders |  |
| Q1 | 5.3 (5.2, 5.5) |
| Q2 | 3.5 (3.4, 3.7) |
| Q3 | 2.7 (2.6, 2.8) |
| Q4 | 2.3 (2.1, 2.5) |
| Q5 | 1.8 (1.6, 1.9) |
| Personality disorders |  |
| Q1 | 6.7 (6.5, 7.0) |
| Q2 | 4.7 (4.5, 4.9) |
| Q3 | 3.7 (3.5, 3.9) |
| Q4 | 3.3 (3.1, 3.5) |
| Q5 | 2.6 (2.4, 2.7) |
| Broadly defined schizophrenia |  |
| Q1 | 3.9 (3.7, 4.0) |
| Q2 | 2.4 (2.3, 2.5) |
| Q3 | 2.0 (1.9, 2.1) |
| Q4 | 1.8 (1.7, 2.0) |
| Q5 | 1.6 (1.5, 1.7) |
| Mood disorders |  |
| Q1 | 9.6 (9.3, 9.9) |
| Q2 | 7.8 (7.6, 8.0) |
| Q3 | 6.7 (6.5, 7.0) |
| Q4 | 5.9 (5.7, 6.1) |
| Q5 | 5.6 (5.4, 5.9) |
| Anxiety/somatoform disorders |  |
| Q1 | 15.8 (15.5, 16.2) |
| Q2 | 12.1 (11.8, 12.4) |
| Q3 | 10.6 (10.3, 11.0) |
| Q4 | 9.9 (9.5, 10.2) |
| Q5 | 8.4 (8.0, 8.7) |
| Eating disorders |  |
| Q1 | 1.6 (1.5, 1.7) |
| Q2 | 1.3 (1.3, 1.4) |
| Q3 | 1.5 (1.4, 1.6) |
| Q4 | 1.4 (1.3, 1.5) |
| Q5 | 1.6 (1.5, 1.7) |

Values indicate the absolute risk of being diagnosed with the disorder of interest by age 37 years. Q=quintile.
